# DNA damage response signatures are associated with frontline chemotherapy response and routes of tumor evolution in extensive stage small cell lung cancer

**DOI:** 10.1186/s12943-025-02291-0

**Published:** 2025-03-20

**Authors:** Benjamin B. Morris, Simon Heeke, Yuanxin Xi, Lixia Diao, Qi Wang, Pedro Rocha, Edurne Arriola, Myung Chang Lee, Darren R. Tyson, Kyle Concannon, Kavya Ramkumar, C. Allison Stewart, Robert J. Cardnell, Runsheng Wang, Vito Quaranta, Jing Wang, John V. Heymach, Barzin Y. Nabet, David S. Shames, Carl M. Gay, Lauren A. Byers

**Affiliations:** 1 Department of Thoracic/Head and Neck Medical Oncology, The University of Texas MD Anderson Cancer Center, 1515 Holcombe Blvd, Houston, TX 77030 USA; 2 Department of Bioinformatics and Computational Biology, The University of Texas MD Anderson Cancer Center, Houston, TX USA; 3 Medical Oncology Department, Vall d’Hebron University Hospital, Barcelona, Spain; 4 Medical Oncology Department, Hospital del Mar, Barcelona, Spain; 5 Department of Oncology Biomarker Development, Genentech Inc, South San Francisco, CA USA; 6 Department of Pharmacology, Vanderbilt University School of Medicine, Nashville, TN USA; 7 Department of Hematology/Oncology, The University of Texas MD Anderson Cancer Center, Houston, TX USA; 8 Department of Biochemistry, Vanderbilt University School of Medicine, Nashville, TN USA

## Abstract

**Introduction:**

A hallmark of small cell lung cancer (SCLC) is its recalcitrance to therapy. While most SCLCs respond to frontline therapy, resistance inevitably develops. Identifying phenotypes potentiating chemoresistance and immune evasion is a crucial unmet need. Previous reports have linked upregulation of the DNA damage response (DDR) machinery to chemoresistance and immune evasion across cancers. However, it is unknown if SCLCs exhibit distinct DDR phenotypes.

**Methods:**

To study SCLC DDR phenotypes, we developed a new DDR gene analysis method and applied it to SCLC clinical samples, *in vitro*, and *in vivo* model systems. We then investigated how DDR regulation is associated with SCLC biology, chemotherapy response, and tumor evolution following therapy.

**Results:**

Using multi-omic profiling, we demonstrate that SCLC tumors cluster into three DDR phenotypes with unique molecular features. Hallmarks of these DDR clusters include differential expression of DNA repair genes, increased replication stress, and heightened G2/M cell cycle arrest. SCLCs with elevated DDR phenotypes exhibit increased neuroendocrine features and decreased “inflamed” biomarkers, both within and across SCLC subtypes. Clinical analyses demonstrated treatment naive DDR status was associated with different responses to frontline chemotherapy. Using longitudinal liquid biopsies, we found that DDR Intermediate and High tumors exhibited subtype switching and coincident emergence of heterogenous phenotypes following frontline treatment.

**Conclusions:**

We establish that SCLC can be classified into one of three distinct, clinically relevant DDR clusters. Our data demonstrates that DDR status plays a key role in shaping SCLC phenotypes and may be associated with different chemotherapy responses and patterns of tumor evolution. Future work targeting DDR specific phenotypes will be instrumental in improving patient outcomes.

**Supplementary Information:**

The online version contains supplementary material available at 10.1186/s12943-025-02291-0.

## Introduction

Small cell lung cancer (SCLC) is a high-grade neuroendocrine malignancy and the most lethal form of lung cancer [1]. This poor prognosis is due in large part to SCLC’s recalcitrance to therapy. Unlike most non-small cell lung cancers (NSCLCs), SCLCs lack targetable oncogenic driver alterations and instead develop through dual inactivation of *TP53* and *RB1* tumor suppressor genes [2, 3]. In lieu of oncogene-directed targeted therapies, a chemotherapy doublet of etoposide and platinum-based chemotherapy (EP) is the backbone of SCLC frontline therapy. While most SCLCs are initially exquisitely sensitive to chemotherapy, most tumors recur and rapidly progress as chemoresistant disease soon after initial treatment [1]. The addition of anti-PDL1 immunotherapy to EP chemotherapy has improved outcomes for extensive stage (ES) SCLC patients [4]. However, frontline chemoimmunotherapy does not produce durable disease control for the vast majority of ES-SCLC patients. Identifying and characterizing biologic programs potentiating chemoresistance and immune evasion is a crucial unmet need in SCLC.

Research from our group and others has demonstrated that transcriptional profiling can identify clinically relevant, molecularly distinct subtypes of SCLC [5–7]. Using unsupervised clustering and large clinical datasets, Gay et al. found that SCLC is composed of four distinct subtypes marked by expression of lineage-specific transcription factors or characterized by “inflamed” biomarkers [5]. Clinically, “inflamed” tumors, which account for roughly 20% of de novo SCLC, tend to derive more durable benefit from frontline chemoimmunotherapy. Despite this knowledge, it is unknown why the overwhelming majority of ES-SCLCs do not respond durably following frontline treatment.

Studies have shown that increased expression of DNA repair genes is associated with chemotherapy resistance and immune cold tumor microenvironments across cancers [8–12]. In SCLC, recent studies have identified an inverse correlation between DNA repair gene expression and “inflamed” biomarkers in relapsed tumors [13]. However, it is unknown if SCLCs exhibit distinct DNA damage response (DDR) phenotypes and if these phenotypes identify tumors with unique molecular features not currently captured by SCLC subtypes alone.

We hypothesized that differential regulation of the DDR machinery would identify biologically distinct, clinically relevant clusters of SCLC tumors. To test our hypothesis, we developed a new DDR gene expression analysis method and applied it to SCLC clinical samples, *in vitro*, and *in vivo* SCLC model systems. Using this method, we investigated how expression of the DDR machinery is associated with neuroendocrine biology, immune evasion, frontline chemotherapy response, and tumor evolution in SCLC.

## Materials and methods

### Molecular profiling data

We analyzed bulk RNA sequencing data for SCLC tumor samples from the MD Anderson GEMINI cohort [14], the IMpower133 clinical trial [4, 7], SCLC cell lines [15], and SCLC and high grade neuroendocrine (hgNEC) patient derived xenograft (PDX) and circulating tumor cell derived xenograft (CDX) models. Single cell RNA sequencing (scRNAseq) from these PDX/CDX models was also analyzed. SCLC cell line reverse phase proteomic array (RPPA) protein expression data was previously generated by our group and the Broad Institute [15, 16]. SCLC cell line whole exome sequencing mutation data was previously generated by the Broad Institute [17]. GEMINI, IMpower133, and SCLC PDX/CDX RNA sequencing data are presented as normalized transcripts per million (TPM) values. SCLC cell line gene expression data are presented as z-scores, as deposited by Tlemsani et al. [15]. RPPA data are presented as normalized, log2 transformed values. Reduced representation bisulfite sequencing (RRBS) methylation data analyzed in this study were previously generated by Heeke et al. [14].

### Weighted Expression Score (WE Score) analysis method

WE scores were calculated to evaluate transcriptional regulation of ten distinct DDR pathways in SCLC samples (Supplementary Fig. 1A). Gene expression z-scores were calculated for 130 DDR genes functioning across the 10 pathways. Z-score values were used to prevent differences in transcript abundance across genes from skewing pathway regulation scores. WE scores were generated by multiplying gene expression z score values by gene-specific essentiality scaling factor (ESF) values to account for effector importance to pathway function (Supplementary Fig. 1B). ESF values were assigned following extensive literature review and are based on DDR gene essentiality in genetic knockout studies and familial cancer syndromes [18–27]. DDR gene ESF values are listed in Supplementary Table 1. These products were summed and divided by the total number of genes in each pathway to generate raw WE scores. Raw WE scores were then scaled across samples. The fviz_nbclust function in the factoextra R package was used to determine the optimal number of *k* clusters by minimizing the total within sum of squares variance between clusters. A maximum of k = 10 clusters were considered. Samples were clustered using the ComplexHeatmap R package and the statistically defined optimal number of *k* clusters.

### SCLC subtype assignment

IMpower133 tumor samples and SCLC cell lines were subtyped using our previously described non-negative matrix factorization (NMF) approach and 1300 gene signature [5]. GEMINI tumor samples and SCLC PDX/CDX models were subtyped using our new SCLC Gene-Ratio Classifier (SCLC-GRC) [14]. As our SCLC-GRC method was trained using IMpower133 samples, this approach could not be used to subtype IMpower133 samples. In addition to these subtyping approaches, IMpower133 samples were also subtyped using Genentech’s recent NMF approach [7].

### Differential gene expression and quantitative set analysis for gene expression (QuSAGE)

Differential gene expression and quantitative set analysis for gene expression (QuSAGE) was used to identify transcriptional hallmarks of IMpower133 DDR clusters. Genes differentially expressed between DDR clusters were identified using pairwise comparisons and the limma R package [7, 28]. Gene set analyses were performed using the QuSAGE R package [7, 29].

### Immune and stromal cell infiltration estimation

The tidyestimate R package was used to estimate bulk immune and stromal cell infiltration in GEMINI and IMpower133 samples [30].

### RepStress and Neuroendocrine Score (NE Score) analyses

RepStress and Neuroendocrine Scores were calculated as described previously [11, 31].

### scRNAseq analysis

SCLC PDX/CDX tumors were processed and sequenced using the 10X genomics platform as described previously [32]. The Cell Ranger v3.0.1 pipeline was used to process raw reads to UMI counts [33]. Samples from different sequencing batches were integrated and normalized using Seurat’s SCT integration pipeline [34]. Cells expressing less than 200 genes or those with mitochondrial reads accounting for > 20% of total reads were removed. Principal component analysis (PCA) and UMAP transformations were used for dimensionality reduction with Seurat. Cell cycle states were determined using Seurat’s “CellCycleScoring” function. Binary expression plots were generated using in-house R scripts.

### RNA velocity analysis

scRNAseq data from PDX model SC53 were assessed for stable and transient states based on RNA velocity using scvelo and cellrank Python packages [35–37]. Cells were filtered for quality as described previously [5]. The top 2000 highly variable genes were selected and used for downstream analyses. PCA was applied and the first 30 components were used to identify the 30 nearest neighbors of each cell (kNN). The latent gene space was subjected to t-distributed stochastic network embedding (t-SNE) and clusters were identified using the Leiden algorithm. A model of transcriptional dynamics of splicing kinetics is solved in a likelihood-based expectation maximization by estimating transcriptional state and cell-internal latent time to learn the unspliced/spliced phase trajectory for each gene. This was performed using scvelo’s recover_dynamics in stochastic mode. Driver genes for transitions across states (Leiden clusters) were detected by their high likelihoods in the dynamic model. Random walk simulations were performed to mimic temporal transitions and likely resultant end states using 100 cells randomly placed within each Leiden cluster and proceeding until the system reached steady state. The WEB-based Gene SeT AnaLysis Toolkit (WebGestalt) platform was used to conduct over-representation analyses in order to identify biological processes significantly upregulated in single cell cluster 8 [38].

### Subtype switching analysis

Patient matched longitudinal liquid biopsy samples were subtyped using circulating tumor DNA methylation profiles and our new SCLC DNA methylation classifier (SCLC-DMC) [14]. Patients whose disease progression liquid biopsy was classified as a different SCLC subtype compared to their matched treatment naïve liquid biopsy were deemed to have ‘subtype switched’ following frontline chemoimmunotherapy.

### Statistical analyses

Statistical and bioinformatic analyses were performed in R version 4.2. Wilcoxon tests were used to compare gene expression and protein expression data across DDR clusters. All *p*-values are derived from Wilcoxon tests unless otherwise specified. Boxplots visualize data median as well as 1st and 3rd interquartile ranges. Median overall survival (OS) and progression free survival (PFS) were calculated using the Kaplan–Meier method. The IMpower133 trial was not powered to detect statistically significant differences in molecular subtypes within trial arms. Chi-square tests were used to test for significant enrichment patterns when comparing SCLC subtype and DDR cluster assignments. Chi-square tests were also performed with simulation (2000 replicates) to account for small *n* across some assignment categories. Paired Wilcoxon tests were used to compare promoter methylation and circulating tumor DNA fraction values for our patient-matched baseline and progression liquid biopsy cohort. *p*-values < 0.05 were considered statistically significant.

## Results

### SCLCs cluster into three distinct DDR clusters with unique molecular features

**Fig. 1:**
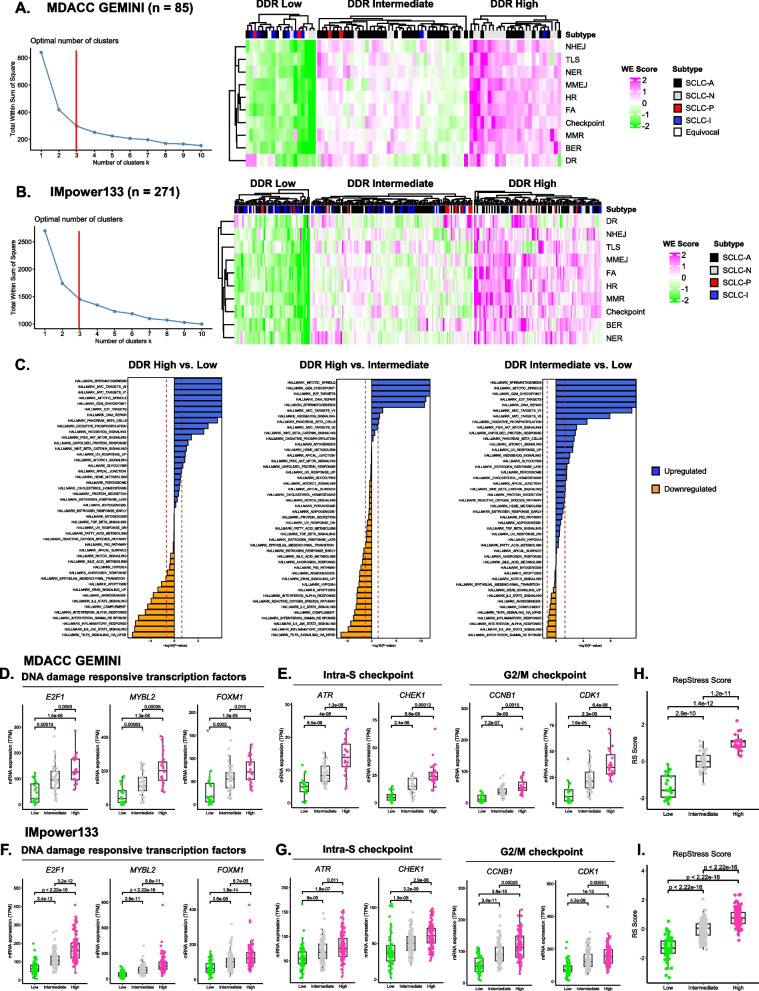
SCLC clusters into three distinct DDR clusters with unique molecular features.** A** MD Anderson GEMINI optimal number of k clusters elbow plot and DDR cluster heatmap. **B** Optimal number of k clusters elbow plot and DDR cluster heatmap for the IMpower133 cohort. **C** IMpower133 DDR cluster differential gene expression and quantitative set analysis (QuSAGE) hallmark pathway results. **D** GEMINI DDR cluster DNA damage responsive transcription factor expression. **E** GEMINI DDR cluster intra-S and G2/M cell cycle checkpoint machinery expression. **F** Expression of DNA damage responsive transcription factors in IMpower133 DDR clusters. **G.** Expression of intra-S and G2/M cell cycle checkpoint machinery in IMpower133 DDR clusters. **H**-**I** GEMINI and IMpower133 DDR cluster RepStress scores. **A-B** NHEJ: Non-homologous end-joining. TLS: Translesion synthesis. NER: Nucleotide excision repair. MMEJ: Microhomology mediated end-joining. HR: Homologous recombination. FA: Fanconi Anemia. Checkpoint: Damage sensing and signaling. MMR: Mismatch repair. BER: Base excision repair. DR: Direct reversal repair

To study SCLC DDR phenotypes, we applied a new DDR analysis method to two SCLC clinical datasets, MD Anderson GEMINI (*n* = 85) and IMpower133 (*n* = 271) (Methods, Supplementary Fig. 1). GEMINI SCLC samples were largely treatment naïve, ES-SCLC tumor samples collected at MD Anderson Cancer Center [14]. IMpower133 samples were exclusively treatment naïve, ES-SCLC tumor samples collected as part of the IMpower133 Phase III clinical trial [4, 7]. Using unsupervised k-means clustering, we identified three statistically defined clusters of GEMINI SCLC tumors demarcated by DDR phenotypes (Fig. 1A). DDR Low tumors were characterized by lowest expression of almost all DDR pathways. DDR Intermediate tumors exhibited a heterogeneous pattern of intermediate to high expression of various DDR pathways. DDR High tumors displayed strong upregulation of most DDR pathways. To validate these findings, we applied our DDR analysis method to an independent dataset of IMpower133 tumor samples. As in our GEMINI dataset, unsupervised k-means clustering again identified three statistically defined clusters of IMpower133 tumors as defined by unique DDR phenotypes (Fig. 1B). Across both datasets, we found a highly similar distribution of samples in each DDR cluster (Supplementary Fig. 2A-B). Inspection of our DDR clusters confirmed that our method indeed identified groups of SCLC tumors with striking differences in individual DDR gene expression patterns (Supplementary Fig. 2C-D). It should be noted that our method clusters tumors with similar global DDR phenotypes by simultaneously evaluating expression of 130 genes functioning across 10 distinct DNA repair pathways. Thus, our method represents a significant divergence from single gene stratification approaches where expression of a single gene is used to separate samples.

We next sought to identify unique molecular features of tumors with differing DDR status. Using QuSAGE analysis, we found that DDR High tumors highly express hallmark pathways controlling DNA repair, E2F transcriptional targets, MYC transcriptional targets, and the G2/M cell cycle checkpoint, compared to DDR Low (Fig. 1C). DDR Low tumors, meanwhile, were characterized by upregulation of pathways controlling apoptosis, interferon alpha signaling, interferon gamma signaling, epithelial to mesenchymal transition (EMT), TNF signaling, immune cell trafficking, and immune cell function, compared to DDR High (Fig. 1C, Supplementary Fig. 3). DDR Intermediate tumors showed evidence of hybrid phenotypes marked by upregulation of DNA repair, cell cycle checkpoint, and inflammatory programs when compared to either DDR High or DDR Low tumors. Collectively, these data indicate that SCLC tumors with different DDR status exhibit a spectrum of DNA repair and inflammatory phenotypes.

Given that gene set enrichment analysis identified E2F transcriptional targets and cell cycle checkpoint pathways as key pathways differentially expressed across DDR clusters, we next investigated expression of individual effectors controlling these processes. Across both GEMINI and IMpower133 datasets, we confirmed that expression of DNA damage responsive transcription factors—including *E2F1*—significantly differed across DDR clusters (Fig. 1D, F). It should be noted that these genes were not included in our DDR analysis method and represent orthogonal validation of unique DDR cluster biology. In addition, we also confirmed that expression of intra-S and G2/M cell cycle checkpoint machinery effectors significantly increased in a DDR specific manner (Fig. 1E, G). As DDR clusters were marked by differential expression of DNA repair and cell cycle checkpoint genes, we hypothesized that SCLC tumors across these clusters experience varying levels of replication stress. Previous studies have demonstrated that the RepStress transcriptional signature reliably identified differences in cellular replication stress and endogenous DNA damage [11]. To test our hypothesis, we calculated RepStress scores for SCLC tumor samples in GEMINI and IMpower133 datasets. As expected, we found that a hallmark of different DDR status is higher levels of replication stress (Fig. 1H, I**)**. Collectively, these data demonstrate that SCLC is composed of three biologically distinct DDR clusters with unique molecular features.

### SCLC DDR clusters are recapitulated in both *in vitro* and *in vivo *model systems

**Fig. 2:**
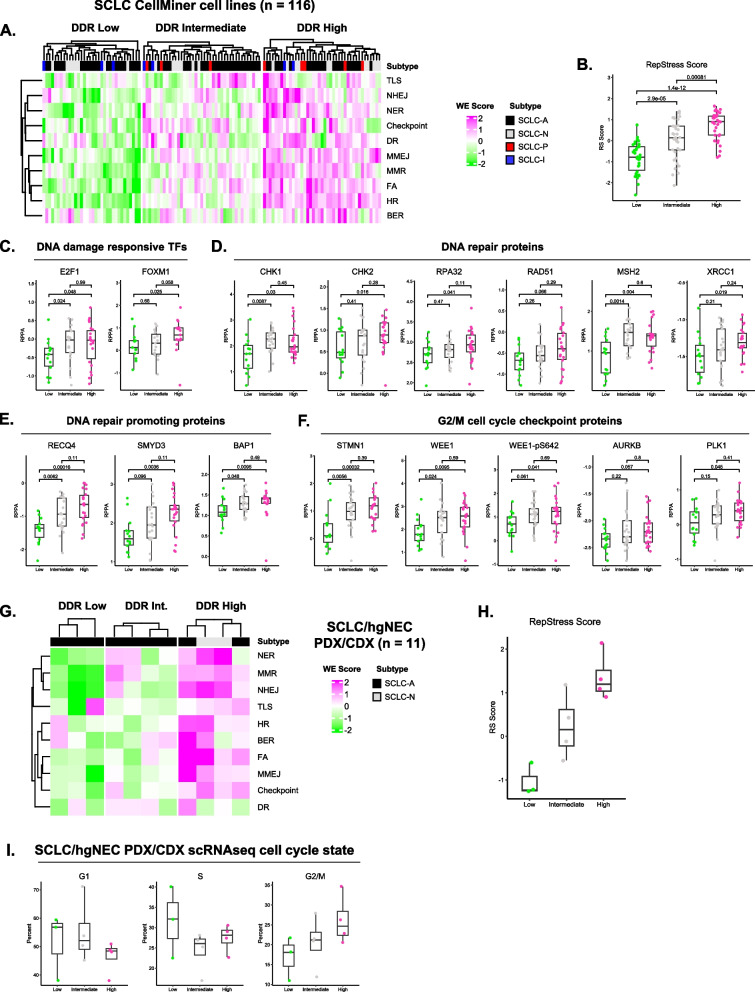
SCLC DDR clusters are recapitulated in both *in vitro* and *in vivo* model systems.** A** DDR cluster heatmap for SCLC CellMiner cell line models. **B** SCLC cell line DDR cluster RepStress scores. **C** SCLC cell line DNA damage responsive transcription factor protein expression. **D** DNA repair protein expression across SCLC cell line DDR clusters. **E** Protein expression of DNA repair promoting effectors across DDR clusters. **F** G2/M cell cycle checkpoint effector protein expression in cell line DDR clusters. **G** DDR cluster heatmap for SCLC/hgNEC PDX and CDX models. **H** SCLC/hgNEC PDX/CDX DDR cluster RepStress scores. **I** SCLC/hgNEC PDX/CDX DDR cluster scRNAseq cell cycle state distribution

To extend our findings, we applied our DDR analysis method to a cohort of SCLC cell line models (*n* = 116) [15]. As in human SCLC samples, unsupervised k-means clustering identified three statistically defined clusters of SCLC cell lines as described by DDR phenotypes (Fig. 2A, Supplementary Fig. 4). We observed a similar distribution of cell line models across DDR clusters, with a slight enrichment of DDR High models compared to that seen in human samples (Supplementary Fig. 5A). Consistent with our previous findings, cell line DDR clusters exhibited differences in DNA damage responsive transcription factor and cell cycle checkpoint machinery transcripts (Supplementary Fig. 5B). Additionally, we observed a dramatic difference in replication stress across cell line DDR clusters (Fig. 2B). Beyond different transcriptional phenotypes, we found that SCLC cell line DDR clusters were marked by significant differences in protein expression. Protein expression of DNA damage responsive transcription factors, DNA damage sensing kinases, and replication stress mediators were significantly different across DDR clusters (Fig. 2C-D). Expression of DNA repair proteins that function in specific repair pathways—MSH2 in MMR and XRCC1 in BER—also increased across DDR clusters. Furthermore, we found expression of proteins that indirectly promote DNA repair also increased across DDR clusters (Fig. 2E). Interestingly, we found no difference in SLFN11 protein expression, which has previously been reported to highly correlate with DNA repair gene expression in SCLC (Supplementary Fig. 5C). Beyond DNA repair proteins, we found that cell line DDR clusters exhibited differential expression of key cell cycle checkpoint regulators (Fig. 2F, Supplementary Fig. 5D). Notably, we observed a concerted upregulation of multiple proteins controlling G2/M cell cycle arrest following DNA damage in DDR High and Intermediate clusters compared to DDR Low (Fig. 2F). This differential expression of G2/M checkpoint proteins was independent of *MYC* expression (Supplementary Fig. 5E). Lastly, we analyzed SCLC cell line whole exome sequencing data to assess potential differences in mutations in key DNA repair genes (Supplementary Fig. 6). As expected, all three DDR groups exhibited near ubiquitous mutational inactivation of *TP53* and *RB1* tumor suppressor genes—known drivers of SCLC tumorigenesis. However, we did not identify statistically significant differential mutational inactivation in any of the 130 DDR genes used in our method (Supplementary Table 2). Additionally, DDR groups did not exhibit differences in tumor mutational burden (Supplementary Fig. 7).

We next applied our DDR analysis method to SCLC PDXs/CDXs. This analysis confirmed that SCLC PDX/CDX models also exhibit three distinct DDR phenotypes (Fig. 2G). Like human SCLC and cell lines, PDX/CDX DDR clusters were marked by increasing levels of key transcripts and differing levels of replication stress (Supplementary Fig. 8A, Fig. 2H). Given that DDR clusters exhibit increased replication stress and markers of G2/M cell cycle arrest, we hypothesized that tumors with different DDR status have different cell cycle state distributions. Using scRNAseq data, we found that increasing DDR status identifies tumors with increasing levels of single tumor cells in G2/M *in vivo* (Fig. 2I, Supplementary Fig. 8B). These data demonstrate that SCLC DDR clusters and their unique molecular features are robustly recapitulated in *in vitro* and *in vivo* model systems.

### SCLC DDR status is significantly associated with SCLC subtypes and neuroendocrine features

**Fig. 3:**
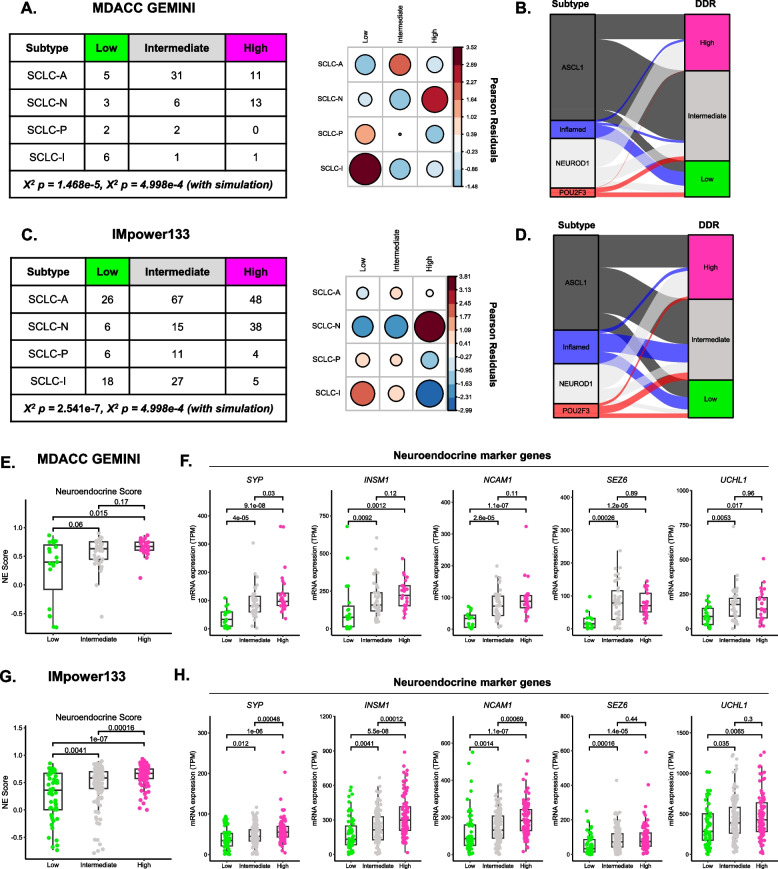
SCLC DDR status is significantly associated with SCLC subtypes and neuroendocrine features.** A** GEMINI DDR cluster and SCLC subtype assignment table where values listed represent number of patients. *X*^2^ Pearson residual dot plot comparing DDR cluster and SCLC subtype assignments for GEMINI samples. **B** Alluvial plot of GEMINI DDR cluster composition by SCLC subtype. **C** IMpower133 DDR cluster and SCLC subtype assignment table. Table values represent number of patients. *X*^2^ Pearson residual dot plot comparing DDR cluster and SCLC subtype assignments for IMpower133 samples. **D** Alluvial plot of IMpower133 DDR cluster composition by SCLC subtype. **E** GEMINI DDR cluster neuroendocrine score analysis. **F** GEMINI DDR cluster neuroendocrine and non-neuroendocrine marker gene expression. **G** IMpower133 DDR cluster neuroendocrine score analysis. **H** IMpower133 DDR cluster neuroendocrine and non-neuroendocrine marker gene expression

Recent studies have shown that SCLC is composed of four subtypes defined by expression of lineage-specific transcription factors or “inflamed” biomarkers [5, 7]. The relationship between these established subtypes and DDR status is unknown. In both GEMINI and IMpower133 datasets, we found DDR status was significantly associated with SCLC subtypes (Fig. 3A-D). All DDR clusters were composed of tumors from multiple subtypes, with individual subtypes being significantly enriched across different DDR clusters (Fig. 3A, C). Specifically, SCLC-N tumors were enriched in DDR High, SCLC-A in DDR Intermediate, and SCLC-P and SCLC-I in DDR Low. An additional analysis recapitulated these findings when using Genentech SCLC subtypes as recently described by Nabet et al. [7] (Supplementary Fig. 9). Using this independent subtyping approach, we confirmed that DDR status was indeed significantly associated with SCLC subtypes. As before, we found that SCLC-N tumors were significantly enriched in DDR High. SCLC-I-nNE tumors, on the other hand, were significantly enriched in DDR Low and significantly underrepresented in DDR High.

Historically, SCLC tumors have been broadly classified based on the presence or absence of neuroendocrine features [31, 39, 40]. To test if DDR status was associated with differing levels of neuroendocrine features, we analyzed neuroendocrine scores which capture expression of lung-specific neuroendocrine and non-neuroendocrine markers [31]. Across both GEMINI and IMpower133 cohorts, we found that NE scores significantly increased across DDR clusters (Fig. 3E, G, Supplementary Fig. 10). This DDR-specific association with neuroendocrine features was further demonstrated by increased expression of individual neuroendocrine marker genes (Fig. 3F, H). We further confirmed these findings in both cell line and PDX/CDX models (Supplementary Fig. 11). Taken together, these data demonstrate that DDR status is significantly associated with SCLC subtypes and neuroendocrine features.

### DDR status identifies a spectrum of “inflamed” features within and across SCLC subtypes

**Fig. 4:**
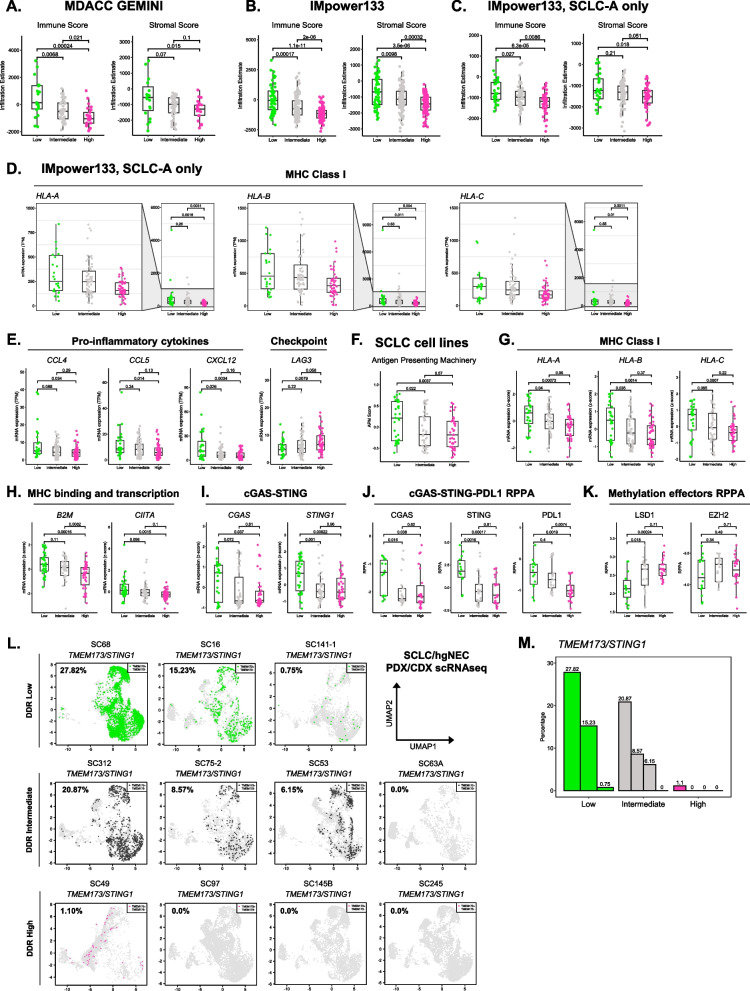
DDR status identifies a spectrum of “inflamed” features both within and across SCLC subtypes.** A** GEMINI DDR cluster ESTIMATE Immune and Stromal scores. **B** IMpower133 DDR cluster Immune and Stromal scores. **C** IMpower133 SCLC-A only DDR cluster Immune and Stromal scores. **D** IMpower133 SCLC-A only DDR cluster MHC Class I gene expression. **E** IMpower133 SCLC-A DDR cluster pro-inflammatory cytokine and immune suppressive checkpoint effector gene expression. **F** SCLC cell line DDR cluster Antigen Presenting Machinery scores. **G** SCLC cell line DDR cluster MHC Class I gene expression. **H** SCLC cell line DDR cluster *B2M* and *CIITA* gene expression. **I.** SCLC cell line DDR cluster *cGAS* and *STING1* gene expression. **J** SCLC cell line DDR cluster cGAS, STING1, and PDL1 protein expression. **K** SCLC cell line DDR cluster LSD1 and EZH2 protein expression. **L.** SCLC/hgNEC PDX/CDX DDR cluster single cell RNAseq *STING1* binary expression plots. **M** SCLC/hgNEC PDX/CDX DDR scRNAseq *STING1* binary expression summary plot

When inspecting DDR signature associations with SCLC subtypes, we were struck by the significant depletion of SCLC-I tumors in DDR High clusters (Fig. 3, Supplementary Fig. 9). We hypothesized that DDR status also identified tumors with a spectrum of “inflamed” features, both within and across SCLC subtypes. As established by Gay et al., a hallmark of SCLC-I tumors is increased immune cell infiltration [5]. Using the ESTIMATE deconvolution algorithm, we found that immune cell and stromal cell infiltration signals significantly decreased across treatment naïve DDR clusters (Fig. 4A-B) [30]. These findings agree well with our previous observation that pathways controlling inflammatory responses, antigen presentation, immune cell trafficking, and immune cell function are strongly anti-correlated with elevated DDR signatures (Fig. 1C, Supplementary Fig. 3). In further support of our findings, recent analyses have also demonstrated that inflamed tumors can be found within each SCLC subtype [7]. To test if our observation was independent of the more infiltrated SCLC-I subtype, we restricted our analyses to SCLC-A tumors which are the most prevalent subtype of SCLC and are a more neuroendocrine, “immune cold” subset of SCLC [5, 41]. Strikingly, we found that splitting treatment naïve SCLC-A tumors only by DDR status identified tumors with significantly different levels of immune and stromal cell infiltration signals (Fig. 4C). Beyond immune cell infiltration, we analyzed other known biomarkers of “inflamed” tumors. Our analysis demonstrated that expression of MHC class I and pro-inflammatory cytokine genes regulating cytotoxic immune cell and antigen presenting cell recruitment decreased across DDR clusters within treatment naïve SCLC-A tumors (Fig. 4D-E). Additionally, we found that expression of immune checkpoint marker *LAG3* increased with increasing DDR status (Fig. 4E). Further analyses demonstrated that other immune checkpoint markers such as *CD274 (PD1)*, *CTLA4*, or *HAVCR2 (TIM3*) were not differentially expressed within SCLC-A tumors when stratified by DDR status (Supplementary Fig. 12).

To confirm that these observations were independent of tumor purity, we repeated our analyses in SCLC cell lines. This analysis confirmed that pure SCLC tumor cells had significantly decreased antigen presenting machinery expression when stratified by DDR status (Fig. 4F-H). This decreased MHC Class I expression was correlated with decreased expression of *CIITA*, a transcription factor responsible for driving MHC Class I gene transcription [42]. Studies have shown that expression of the MHC Class I locus can be positively regulated by inflammatory signaling through the cGAS-STING pathway [43]. Given this, we next investigated if cGAS-STING pathway dysregulation was coincident with MHC Class I silencing in DDR clusters. We found that DDR High and Intermediate models had significantly decreased expression of both *cGAS* and *STING1*, compared to DDR Low (Fig. 4I). This difference was confirmed at the protein level whereas DDR High and Intermediate models had significantly decreased expression of cGAS, STING, and immunomodulatory PDL1 proteins (Fig. 4J). In addition to cGAS-STING dysregulation, we found that protein expression of the epigenetic regulator LSD1 increased in a DDR specific manner and was correlated with MHC Class I silencing, consistent with recent reports (Fig. 4K) [6, 44, 45]. Lastly, we analyzed scRNAseq data from SCLC PDX/CDX models to confirm that these differences were also observed in SCLC tumor cells. As expected, we found that expression of *TMEM173/STING1* decreased across DDR clusters in single tumor cells (Fig. 4L-M). A similar trend was also observed for MHC Class I genes (Supplementary Fig. 13). Collectively, these data robustly demonstrate that DDR status identifies a spectrum of “inflamed” biology both within and across known SCLC subtypes.

### Association between DDR status and frontline chemotherapy response

**Fig. 5:**
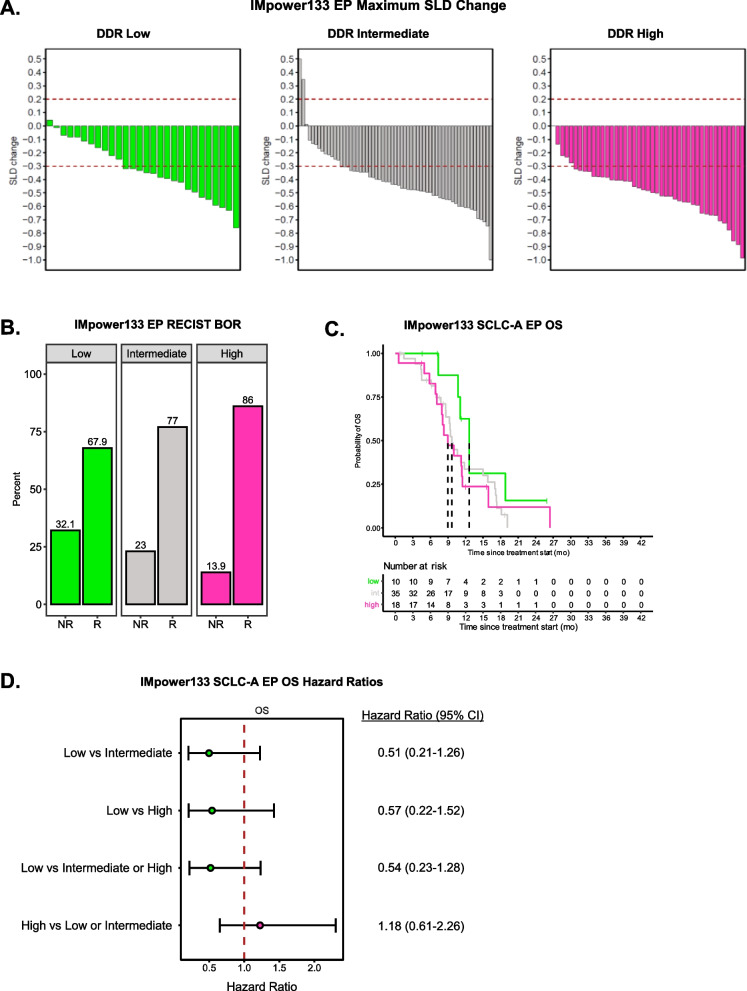
Association between DDR status and frontline chemotherapy response.** A** Maximum sum of longest dimensions (SLD) change for IMpower133 DDR clusters following frontline EP chemotherapy. **B** RECIST Best Overall Response (BOR) for IMpower133 DDR clusters following frontline EP chemotherapy. NR: Non-responder (progressive disease + stable disease). R: Responder (partial response + complete response). **C** Kaplan Meier plot for IMpower133 SCLC-A DDR cluster OS outcomes following frontline EP chemotherapy. **D** Forrest plot for IMpower133 SCLC-A DDR cluster OS outcomes following frontline EP chemotherapy

Given that SCLC DDR clusters exhibited striking differences in DNA repair and replication stress phenotypes, we hypothesized that DDR status would be associated with different responses to chemotherapy in the clinic. To assess the effect of DDR status on frontline chemotherapy response, we analyzed data from the etoposide + carboplatin arm of the IMpower133 Phase III clinical trial [4]. Interestingly, we found that DDR status trended with different depth of response following frontline chemotherapy, as measured by RECIST criteria [46]. DDR High tumors trended toward strongest depth of initial response, measured by RECIST maximum SLD change, followed by DDR Intermediate, and DDR Low tumors (Fig. 5A). This difference in initial tumor shrinkage was recapitulated in differences in RECIST best overall response. Rates of response to frontline chemotherapy were increased in tumors with DDR Intermediate and High phenotypes, compared to DDR Low (Fig. 5B, Supplementary Fig. 14). Conversely, DDR Low tumors were enriched for patients whose tumors did not achieve partial or complete radiographic responses. Paradoxically, patients with DDR High and Intermediate disease had numerically shorter OS and PFS, despite experiencing strongest initial tumor reduction following frontline chemotherapy (Fig. 5C, Supplementary Figs. 14–15). This difference was more pronounced within SCLC-A tumors, where patients with DDR Low tumors had a median OS of 12.65 months (Low vs. Intermediate or High HR = 0.54 (95% CI 0.23–1.28)), compared to 9.85 months for DDR Intermediate, and only 9.49 months in DDR High (Fig. 5D). These findings warrant further exploration in future studies and larger patient cohorts.

### DDR status is linked to subtype switching and unique routes of disease progression following frontline chemoimmunotherapy

**Fig. 6:**
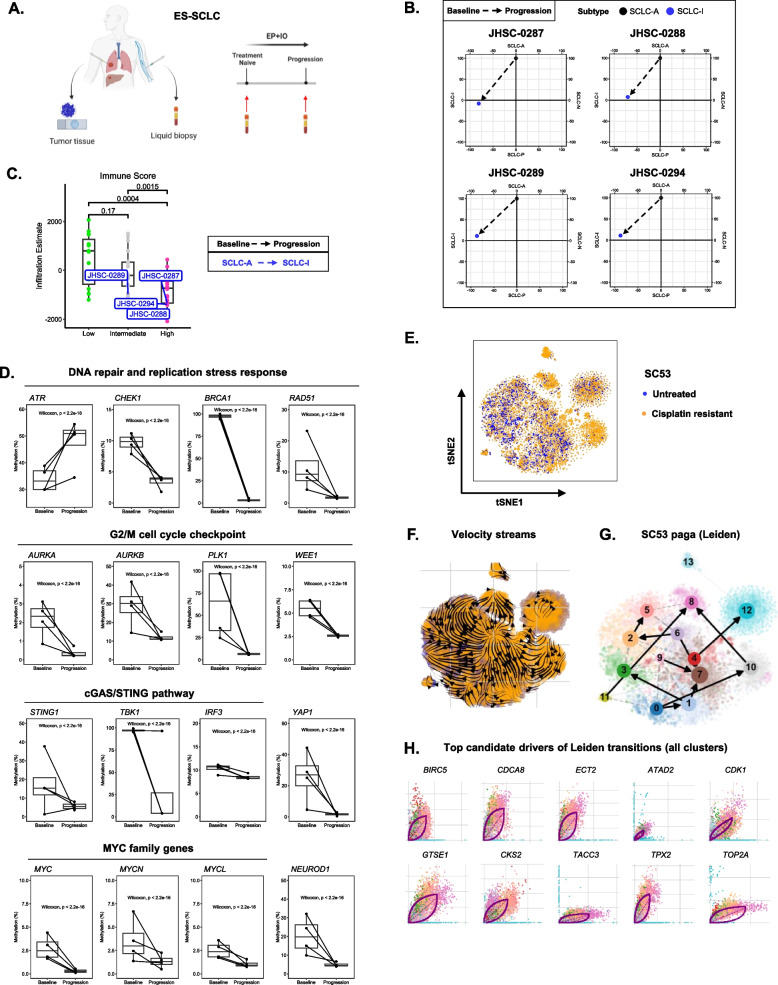
DDR status is linked to subtype switching and unique routes of disease progression following frontline chemoimmunotherapy.** A** ES-SCLC patient matched tissue and longitudinal liquid biopsy collection schema. **B** Subtype switching plots following frontline chemoimmunotherapy. **C** Immune cell infiltration levels in diagnostic tissue samples from subtype switching patients shown in (**B**). **D** Promoter methylation changes in subtype switching patients from baseline to progression on frontline chemoimmunotherapy. **E.** SC53 untreated and cisplatin relapsed scRNAseq tSNE. **F** SC53 untreated and cisplatin relapsed RNA velocity streams. **G** SC53 untreated and cisplatin relapsed Leiden clusters. **H** Top candidate drivers of cell transitions between Leiden clusters

Recent studies have demonstrated that SCLCs can ‘switch’ subtypes and evolve to more ‘inflamed’ phenotypes following therapy [14, 32]. Given that SCLC DDR status is linked to distinct tumor microenvironments at baseline, we hypothesized that initial DDR status would influence tumor evolution following frontline chemoimmunotherapy. To test our hypothesis, we analyzed matched treatment naïve tumor tissue, treatment naïve plasma, and plasma collected at disease progression at first recurrence for six ES-SCLC patients (Fig. 6A). Of these six samples, four tumors underwent subtype switching following frontline therapy—shifting from an SCLC-A state at baseline to an “inflamed” state at progression (Methods, Fig. 6B, Supplementary Fig. 16). Strikingly, patients with inflamed subtype switching tumors did not receive durable benefit from frontline chemoimmunotherapy, with OS and PFS outcomes inferior to that reported for patients with de novo SCLC-I tumors (Supplementary Fig. 17) [5]. All four of these subtype switching tumors were either DDR Intermediate or DDR High. When analyzing these samples further, we found these plastic tumors had some of the lowest initial immune infiltrates before treatment across a third independent cohort of treatment naïve SCLC tumors (Fig. 6C) [14]. These data suggests that DDR-specific immune cell poor microenvironments may be linked to the ability of SCLC cells to progress to an “inflamed” state but not receive durable benefit from frontline chemoimmunotherapy.

To better understand evolving phenotypes of these plastic tumors, we analyzed ctDNA methylation data from matched treatment naïve and progression liquid biopsies. This analysis demonstrated that subtype switching tumors exhibit bifurcated phenotypes enforcing both 1) DNA repair and cell cycle arrest and 2) cGAS-STING, YAP1 related inflammatory programs (Fig. 6D). Specifically, we observed substantial decreases in promoter methylation of genes mediating DNA repair and replication stress responses, translesion synthesis polymerases, G2/M cell cycle arrest, and cGAS-STING signaling (Fig. 6D, Supplementary Fig. 18). We also found decreased methylation of *YAP1*, reminiscent of previous studies demonstrating a *MYC-NEUROD1-YAP1* axis driving plasticity in SCLC (Fig. 6D) [47]. Interestingly, we did not find decreased methylation of immune inhibitory checkpoints that could also promote tumor cell survival despite acquiring ‘inflamed’ features (Supplementary Fig. 18). Further analysis demonstrated that there was no statistically significant difference in circulating tumor DNA fraction when comparing baseline and progression samples for subtype switching tumors (Supplementary Fig. 19). Collectively, these data are consistent with a model wherein chemotherapy induced DNA damage can drive both G2/M cell cycle arrest and cGAS-STING mediated inflammatory signaling responses in SCLC tumor cells.

To validate signals detected in our longitudinal liquid biopsy cohort, we analyzed scRNAseq data from DDR Intermediate PDX model SC53 at both cisplatin sensitive and resistant timepoints (Fig. 6E) [5]. As in human tumors, cells with “inflamed” features emerge following platinum resistance [5]. Using RNA velocity analyses, we found that genes regulating G2/M cell cycle arrest, replication stress, and DNA repair are top drivers of cell plasticity in this model (Fig. 6F-H, Supplementary Figs. 20–22, Supplementary Table 3). Additionally, we identified genes upregulated by cGAS/STING signaling as additional drivers of plasticity (Supplementary Table 3) [48]. While multiple cell states exist in both untreated and cisplatin resistant tumors, random walk simulations demonstrated an overwhelming convergence on Leiden cluster 8, which is characterized by genes regulating G2/M cell cycle arrest and DNA repair—phenocopying our longitudinal liquid biopsy results (Supplementary Fig. 18, Supplementary Figs. 20–22) [38]. Together, these data link baseline DDR status to subtype switching and unique routes of disease progression following frontline therapy.

## Discussion

Small cell lung cancer is a highly aggressive malignancy characterized by recalcitrance to therapy and rapid progression to heterogeneous phenotypes following treatment. In this study, we demonstrate that SCLC tumors cluster into three DDR phenotypes with unique molecular features not captured by subtype assignments alone. Our data show that initial DDR status is associated with chemotherapy response, tumor immune evasion, and evolution to plastic phenotypes following frontline therapy.

Using multi-omic profiling of human SCLC tumor samples, *in vitro* and *in vivo* model systems, we demonstrate that hallmarks of increasing DDR status include increased expression of DNA repair and cell cycle checkpoint effectors, elevated levels of replication stress, and heightened G2/M cell cycle arrest. These data are consistent with a model wherein DDR status identifies differing levels of intrinsic DNA damage and replication stress in treatment naïve SCLC tumors. Despite these striking differences across DDR clusters, we did not find an association with SLFN11 expression, which has been previously reported to positively correlate with DDR gene expression. In fact, our results demonstrate that SLFN11 expression is bimodal within DDR clusters, with each cluster containing both SLFN11 High and Low tumors. These data highlights that our DDR clusters identify SCLCs with similar global DNA repair phenotypes not currently captured by SLFN11 expression alone. Clinically, SLFN11 IHC is being used to try and stratify patients more likely to benefit from DNA damaging targeted therapies [49]. Moving forward, it will be important to consider SLFN11 expression in the context of global DDR status, as it stands to reason that combined assessment of bypass pathways (i.e. translesion synthesis) can further enhance the predictive value of SLFN11 expression.

Beyond DNA damage phenotypes, our results show that increased DDR status is linked with decreased “inflamed” features, both within and across SCLC subtypes. Specifically, we find SCLCs with increased DDR exhibit multiple alterations promoting immune evasion including MHC Class I silencing, decreased pro-inflammatory cytokine expression, and cGAS/STING dysregulation. These alterations are coincident with immune cold microenvironments, as evidenced by decreased immune cell infiltration signatures observed in two independent patient cohorts. Importantly, we demonstrate that these results are independent of the inflamed subtype, whereas stratifying only SCLC-A tumors by their DDR status identifies a spectrum of immune cell infiltration signatures and inflamed biomarker prevalence. Additionally, we establish that these observations are independent of tumor purity by recapitulating our results in pure tumor cell populations both *in vitro* and *in vivo*. It should be noted that DDR specific immune evasive phenotypes are tightly correlated with LSD1 protein expression. Our findings recapitulate reports from several groups linking elevated LSD1 expression with MHC Class I silencing in SCLC [6, 44, 45]. Additionally, our data contextualizes and further extends these findings by linking upregulated LSD1 with elevated replication stress and increased DDR signature expression. Collectively, these data suggest an intriguing model where tumors with increased levels of replication stress are dependent on LSD1-mediated MHC Class I silencing and immune evasion for tumor development and maintenance. Based on these findings, we hypothesize that tumors with elevated DDR may exhibit increased responses to combined LSD1 inhibitor and immunotherapy combinations currently being evaluated in SCLC (NCT05191797). Moving forward, it will be crucial to test whether targeting other immune checkpoints—such as CTLA4, CD276, or LAG3—can better control disease progression in seemingly more immune evasive DDR Intermediate and High tumors.

To our knowledge, our study represents one of the largest analyses of transcriptional markers and frontline chemotherapy responses in ES-SCLC. Our work suggests that treatment naïve DDR status may identify SCLC tumors with differing depth and duration of response to frontline chemotherapy. While underpowered, signals identified in our study generate intriguing hypotheses with respect to the well-recognized paradox of SCLC’s initial exquisite chemotherapy sensitivity and propensity for rapid chemoresistance [1]. Together, our results suggest a model where high levels of replication stress and cell-intrinsic DNA damage drive compensatory upregulation of the DDR machinery in treatment naïve tumors, potentially priming SCLCs for resistance to DNA damaging therapies and ultimately leading to poor patient outcomes. Our finding that DDR High and Intermediate patients have numerically shortened overall survival following frontline chemotherapy, compared to DDR Low, may be explained by the fact that many additional lines of therapy in SCLC are also DNA-damage based. It is possible that as these tumors quickly develop resistance to frontline DNA-damaging therapies, they may be primed for cross-resistance to additional lines of therapy with similar mechanisms of action, a phenomena recently confirmed in SCLC [50]. Interestingly, multi-omic profiling of preclinical SCLC models demonstrated that tumors with increased DDR signatures exhibited elevated expression of the epigenetic regulator SMYD3. Recently, SMYD3 has been shown to drive resistance to alkylating chemotherapeutic agents and that pharmacologic inhibition of SMYD3 reverses resistance in SCLC models [51]. Moving forward, an open question is whether targeting SMYD3 can increase efficacy of lurbinectedin in a DDR specific fashion.

Lastly, our data highlight that treatment naïve DDR status may have implications for SCLC evolution. Using longitudinal liquid biopsies, we find that tumors with elevated DDR become highly plastic and can acquire non-neuroendocrine features following frontline therapy. Paradoxically, these plastic tumors shift subtypes towards a more “inflamed” state but do not derive durable benefit from frontline chemoimmunotherapy targeting the PD1 axis. Additionally, these plastic tumors have some of the most immune cell poor tumor microenvironments at baseline. Given this, we hypothesize that the lack of immune cell infiltration may be necessary for outgrowth of resistant SCLC tumors cells with “inflamed” features that could otherwise be expected to be eliminated by tumor microenvironment resident immune cells. Using longitudinal methylation profiling, we find these tumors upregulate bifurcated phenotypes regulating both DNA repair and inflammatory programs. These data are reminiscent of findings from Lissa et al. where SCLCs with hybrid neuroendocrine and non-neuroendocrine features respond poorly to therapy and rapidly progress [13]. Interestingly, in their report the authors find that these hybrid tumors are resistant to targeted ATR inhibition. In our study, we find that plastic tumors with similar features converge on a G2/M arrest and increasingly methylate *ATR*, providing a potential explanation for the observed lack of response to ATR inhibition. Our results instead argue for targeting these hybrid tumors with agents that inhibit G2/M cell cycle effectors, such as PLK1, AURK, and WEE1 [52]. Furthermore, we find that these plastic tumors upregulate the *MYC*/*NEUROD1*/*YAP1* axis previously shown to drive plasticity in SCLC [47]. Our data extend previous reports by linking *YAP1* upregulation with coincident *cGAS-STING* and inflammatory signaling activity following therapy, providing a potential mechanism for *YAP1* upregulation. We also observed an upregulation of several TLS polymerases at progression in these plastic tumors. Recent work has demonstrated that upregulation of the TLS machinery underpins resistance to a host of DNA damaging agents in SCLC [50]. Our work provides further rationale for exploring targeted TLS inhibitor combinations in relapsed tumors as a mechanism for chemosensitization. It should be noted that we cannot definitively exclude the possibility that these changes in gene methylation may stem from non-tumor compartments, such as immune cells. However, we found no significant difference in ctDNA fraction between paired baseline and progression timepoints for subtype switching patients. These data provide increased confidence that we are detecting signals stemming from SCLC tumor cells across timepoints. Additionally, we detected changes in mRNA expression of genes regulating these same pathways using scRNAseq from pure SCLC tumor cells *in vivo*, before and after chemotherapy. These data confirm that changes we detect in our longitudinal patient samples can indeed emanate from SCLC tumor cells following chemotherapy resistance. Future studies will be needed to dissect the mechanisms underpinning these phenotypic shifts.

Our study has several limitations. First, our study is retrospective in nature. Second, analysis of IMpower133 patient outcomes following frontline chemotherapy was underpowered for biomarker subgroup analyses. Our findings linking DDR phenotypes with different responses to frontline chemotherapy require validation in future studies. Third, we lacked matched genomic profiling data from our human patient cohorts and thus could not determine underlying genetic drivers of distinct DDR phenotypes. Lastly, our analysis of DDR specific subtype switching is constrained by a small sample size. Future studies with larger longitudinal cohorts will be needed to confirm our findings with respect to DDR specific SCLC evolution and phenotypic plasticity.

In conclusion, we establish that SCLC clusters into three biologically distinct, clinically relevant DDR clusters. Our work demonstrates that initial DDR status plays a key role in shaping SCLC phenotypes and may be associated with chemotherapy response and patterns of tumor evolution following frontline therapy. Future work targeting DDR cluster specific phenotypes will be instrumental for ultimately improving SCLC patient outcomes.

## Supplementary Information

Additional file 1: Supplementary Figure 1: DDR subtyping analysis overview. A. DDR network overview. HR: Homologous recombination. MMEJ: microhomology-mediated end-joining. NHEJ: Non-homologous end-joining. BER: Base excision repair. NER: Nucleotide excision repair. MMR: Mismatch repair. DR: Direct reversal repair. TLS: Translesion synthesis. Checkpoint: Damage sensing and signaling. FA: Fanconi Anemia. Numbers in parentheses indicate number of pathway genes analyzed by our method. B. WE score formula and Essentiality Scaling Factor (ESF) criteria. Supplementary Figure 2: DDR cluster prevalence and DDR pathway single gene heatmaps. A. DDR cluster prevalence in GEMINI cohort. B. DDR cluster prevalence in IMPOWER133 cohort. C. GEMINI DDR pathway single gene expression heatmap. D. IMPOWER133 DDR pathway single gene expression heatmap. Supplementary Figure 3: IMpower133 DDR cluster differential gene expression and quantitative set analysis (QuSAGE) BG signature results. Supplementary Figure 4: SCLC CellMiner cell line DDR optimal number of k clusters elbow plot. Supplementary Figure 5: SCLC CellMiner cell line DDR cluster prevalence and marker expression. A. DDR cluster prevalence in SCLC cell line models. B. DNA damage responsive transcription factors, intra-S cell cycle checkpoint, and G2/M cell cycle checkpoint machinery expression across SCLC cell line DDR clusters. C. SLFN11 protein expression in SCLC cell line DDR clusters. D. Total RB1 and RB1-S807.S811 phospho protein expression across SCLC cell line DDR clusters. E. Cell line DDR cluster MYC family gene expression. Supplementary Figure 6: SCLC cell line DDR cluster mutation profiling. Oncoprints for *TP53, RB1*, and DDR gene mutations in DDR Low, Intermediate, and High clusters. Mutation data is from CCLE whole exome sequencing [17]. Supplementary Figure 7: SCLC cell line DDR cluster nonsynonymous tumor mutational burden (TMB). TMB data is from CCLE whole exome sequencing [17]. Supplementary Figure 8: SCLC/hgNEC PDX/CDX DDR gene expression and cell cycle state distribution. A. Expression of DNA damage responsive transcription factors, intra-S, and G2/M cell cycle checkpoint effectors in PDX/CDX DDR clusters. B. PDX/CDX DDR cluster scRNAseq cell cycle state distributions. Supplementary Figure 9: IMpower133 subtyping method comparison. A. Three way alluvial plot demonstrating assignment overlap between MDACC SCLC subtypes [5], Genentech (GNE) subtypes [7], and DDR status. B. IMpower133 DDR cluster and GNE subtype assignment table. Values listed represent the number of samples in each assignment category. The corresponding *X*^2^ Pearson residual dot plot comparing DDR cluster and GNE subtype assignments is shown on the right. Supplementary Figure 10: GEMINI and IMpower133 DDR cluster neuroendocrine score single gene heatmaps. A. GEMINI DDR cluster neuroendocrine score single gene expression heatmap. B. IMpower133 DDR cluster neuroendocrine score single gene expression heatmap. Supplementary Figure 11: SCLC CellMiner DDR cluster neuroendocrine features. A. SCLC cell line DDR cluster NE scores. B. SCLC cell line DDR cluster neuroendocrine status as reported by Tlemsani et al. C. SCLC PDX/CDX DDR cluster NE scores. Supplementary Figure 12: IMpower133 SCLC-A only immune checkpoint marker expression. Boxplots depict mRNA expression of *CD274/PD1, CTLA4, *and *HAVCR2/TIM3* in SCLC-A tumors, split by their DDR status. Supplementary Figure 13: SCLC/hgNEC PDX/CDX DDR cluster MHC Class I scRNAseq expression. Supplementary Figure 14: IMpower133 DDR cluster chemotherapy response analysis. A. RECIST Best Overall Response (BOR) for IMpower133 DDR clusters following frontline EP chemotherapy. PD: Progressive disease. SD: Stable disease. PR: Partial response. CR: Complete response. B. Progression free survival Kaplan Meier plot for SCLC-A DDR clusters following frontline EP chemotherapy. C. Forest plot for progression free survival for SCLC-A DDR clusters following frontline EP chemotherapy. Supplementary Figure 15: IMpower133 DDR cluster all subtypes chemotherapy response analysis. A. Overall survival (OS) Kaplan Meier plot for all subtypes DDR clusters following frontline EP chemotherapy. B. Progression free survival (PFS) Kaplan Meier plot for all subtypes DDR clusters following frontline EP chemotherapy. C. Forrest plots for all subtype DDR cluster OS and PFS outcomes following frontline EP chemotherapy. Supplementary Figure 16: MDACC GEMINI subtype switching plots. Subtype space and SCLC-DMC subtype calls for MDACC GEMINI subtype switching patients. Supplementary Figure 17: MDACC GEMINI subtype switching patient outcomes following frontline chemoimmunotherapy. A. Overall survival outcomes following frontline chemoimmunotherapy. B. Progression free survival following frontline chemoimmunotherapy. Supplementary Figure 18: Promoter methylation changes for MDACC GEMINI subtype switching patients from baseline to progression following frontline chemoimmunotherapy. For all panels, data presented is RRBS methylation data previously published by Heeke et al [14]. Supplementary Figure 19: Circulating tumor DNA fraction for subtype switching tumors at baseline and progression following frontline chemoimmunotherapy. Supplementary Figure 20: SC53 Leiden cluster specific top drivers of plasticity. scRNAseq data for SC53 is from Gay et al. [5]. Supplementary Figure 21: WebGestalt over-representation analysis of SC53 Leiden Cluster 8 marker genes. Supplementary Figure 22: SC53 Leiden cluster specific random walk simulations. scRNAseq data for SC53 is from Gay et al. [5]. Supplementary Table 1: WE score individual DDR gene Essentiality Scaling Factor (ESF) assignments and example raw WE score generation. Supplementary Table 2: SCLC cell line whole exome sequencing mutation enrichment statistics by DDR cluster. Genes not listed in Supplementary Table 2 were not mutated in any DDR Low, DDR Intermediate, or DDR High cell line models. Supplementary Table 3: SC53 dynamic genes by Leiden cluster.

## Data Availability

GEMINI RNA sequencing and methylation profiling data are deposited in dbGAP under accession number phs003416.v1.p1. IMpower133 RNA sequencing and limited clinical data are available by request via the European Genome-Phenome Archive under accession number EGAS50000000138. To request access to this IMpower133 data, researchers should contact devsci-dac-d@gene.com. SCLC cell line RNA sequencing and reverse phase proteomic array (RPPA) data are available through the SCLC CellMiner database (SCLCCellMinerCDB) (https://discover.nci.nih.gov/rsconnect/SclcCellMinerCDB/). SCLC cell line whole exome sequencing data are available through cBioPortal (https://cbioportal.org). SCLC/hgNEC CDX/PDX bulk RNA sequencing and single cell RNAseq data are deposited in GEO under accession number GSE138474. Code generated in this manuscript will be made available upon publication.
